# Doctor, I'm Seeing Two of Everything

**DOI:** 10.7759/cureus.6602

**Published:** 2020-01-08

**Authors:** Alex Davis, Christopher M Lloyd

**Affiliations:** 1 Emergency Medicine, OhioHealth Doctors Hospital, Columbus, USA

**Keywords:** diplopia, vertical diplopia, emergency, emergency, cranial nerve, optic neuropathy, dorsal midbrain, vision change, painless vision change

## Abstract

Acute diplopia is a rare chief complaint with a broad differential diagnosis; key historical and physical characteristics aid with emergency management. This case report discusses the important findings, imaging, and multidisciplinary interaction between emergency medicine, ophthalmology, and neurology regarding the approach to addressing acute painless vertical diplopia. A 51-year-old male presented to the emergency department (ED), reporting that he was seeing 'two of everything,' since awakening. Although the patient had a history of ischemic stroke, he had never experienced this sensation of diplopia. His ED workup was essentially unremarkable; he was admitted for evaluation of the possibilities of a fourth cranial nerve (CN IV) palsy, acute Parinaud syndrome, or ischemic stroke. Ultimately the patient was sent home one day after admission with the diagnosis of CN IV neuropathy. Highlighted is an approach to undifferentiated diplopia with an included discussion of the pathophysiology of a CN IV palsy and Parinaud syndrome. Understanding basic pathophysiology and anatomy allows for a proper history, physical exam, and appropriate consultation. With these tools, emergency physicians can improve their approach to patients with acute diplopia when arriving at the ED.

## Introduction

Acute diplopia is a rare chief complaint that must be well understood to initiate proper diagnostic and treatment modalities. The differential is broad, but management can improve if basic understanding is improved. In a review article completed by Evidence-Based Medicine, neuro-ophthalmological conditions present in two rather distinct ways: visual field defects and diplopia. The former is generally understood as a retinal input condition, while the latter, which will be our focus, affects ocular motility [1]. Parinaud syndrome and fourth cranial nerve (CN IV) palsy manifest as vertical diplopia. Both have key components that with the proper identification of signs and symptoms can allow for differentiation from other pathologies. In a case report completed by Bhola et al., they identified Parinaud syndrome as a collection of signs that include vertical gaze palsy with diplopia, convergence retraction, and light near dissociation [2]. Isolated CN IV palsy can result from trauma, congenital abnormalities, ischemia, or mass compression; however, the final manifestation is vertical diplopia [3]. 

## Case presentation

A 51-year-old man with a history of ischemic stroke without residual deficits, hypothyroidism, and tobacco abuse presented with the concern of acute diplopia that started in the morning upon awakening. The double vision worsened with both upward and downward gaze. He described the vision change as one image stacked on top of another. The last known normal vision was the prior night. In addition, he reported associated nausea when standing. He denied ocular pain, trauma, or other neurological deficits.

On examination, his blood pressure was 127/58, temperature 98.6°F, heart rate 77 beats per minute, and oxygen saturation of 98% on room air. He was awake, alert, and oriented; however, he appeared anxious without acute distress. An eye exam revealed bilateral ocular paralysis with an upward gaze that was worse on the right. His medial and lateral eye movements were intact. His pupils were equal and reactive without afferent pupillary defect. There was no evidence of conjunctival injection, cellulitis, or erythema surrounding the eye. The remainder of his physical examination, including a full neurological exam, was normal. 

A complete blood count (CBC), comprehensive metabolic panel, and prothrombin time/international normalized ratio (PT/INR) were completed, with findings showing a random glucose of 109 mg/dl. The CBC and PT/INR results were otherwise unremarkable. Computed tomography scan without any contrast of the brain demonstrated no intracranial hemorrhage, mass effect, or shift of the midline structures. A bedside ocular ultrasound was completed and showed no evidence for retinal detachment, lens detachment, or vitreous hemorrhage. The optic nerve diameter was <5 mm. Ophthalmology and the stroke neurologist were consulted, and they recommended no immediate interventions; however, admission with further imaging was warranted. At the time of admission, the etiology of the patient’s deficits was unclear with consideration given to ischemia, trauma, mass effect, and congenital abnormalities. However, CN IV palsy or Parinaud syndrome remained at the forefront.

**Figure 1:**
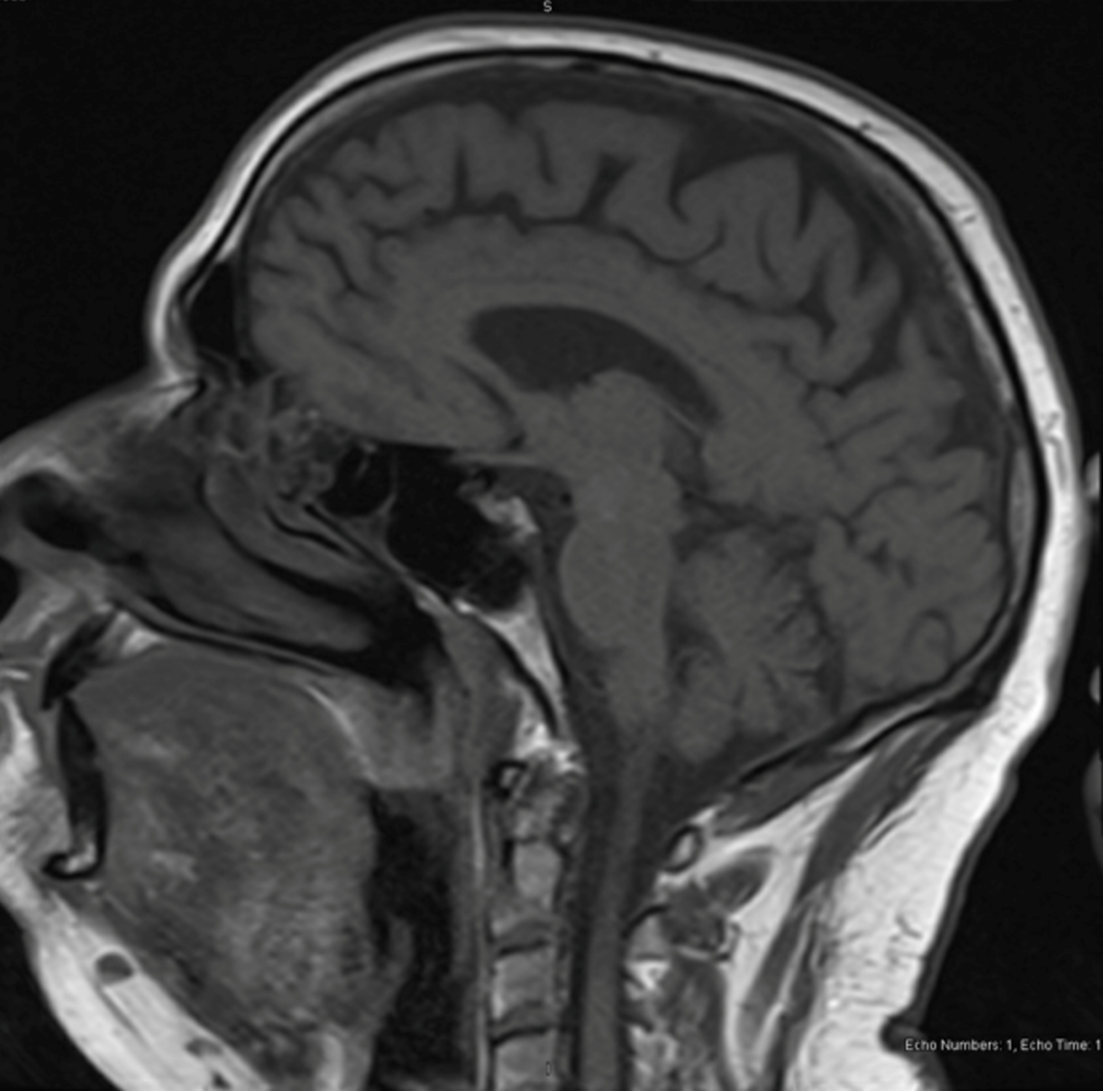
Sagittal MRI after resolution of patient's symptoms with no acute findings

**Figure 2:**
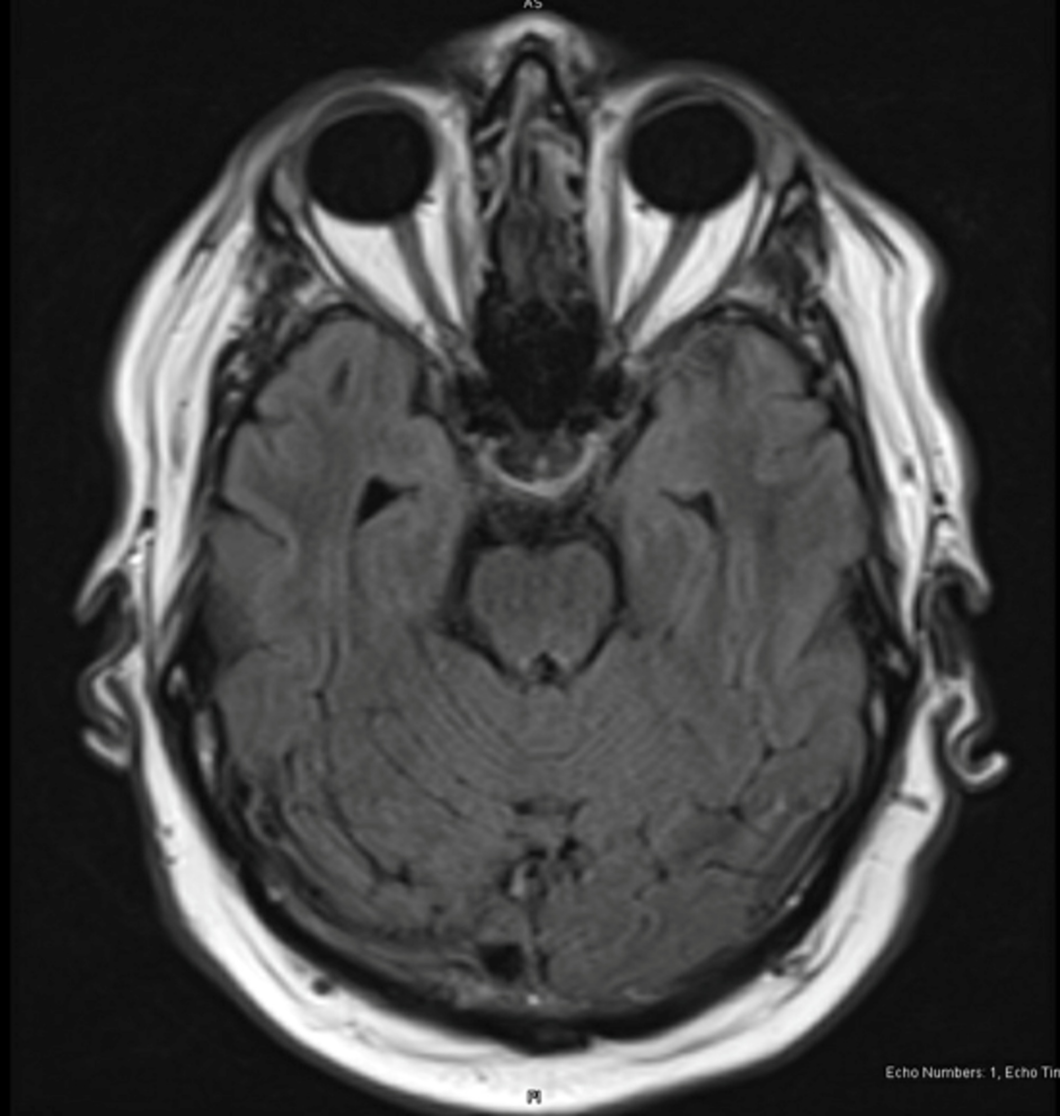
Transverse MRI with no acute findings

The patient’s vertical diplopia improved overnight. The magnetic resonance imaging (MRI) results revealed no acute findings (Figures 1, 2). However, there was evidence of old ischemic changes in the white matter of the cerebral hemispheres and multiple old lacunar infarcts in the right and left cerebellar hemispheres. The patient was evaluated by neurology who attributed this diplopia to an acute non-painful left CN IV neuropathy. They recommended tests for erythrocyte sedimentation rate, creatine phosphokinase, and myasthenia antibodies-all of these were normal. The patient was discharged with outpatient ophthalmology and neurology visits were scheduled.

## Discussion

This case highlights vertical diplopia that was ultimately attributed to an acute left CN IV neuropathy with Parinaud syndrome as an additional concern. Congenital abnormalities and trauma can also lead to isolated CN IV palsies; however, these are beyond the scope of this discussion. Initial concerns in the emergency department (ED) included an ischemic stroke prompting neurological evaluation; however, the patient had no focal findings save for his ocular exam. Urgent ophthalmology consultation allowed for expedited evaluation with concern for either Parinaud syndrome, which could have surgical implications, or a CN IV palsy. The CN IV is found at the pontomesencephalic junction and is unique because it exits dorsally and innervates the contralateral superior oblique muscle due to its early decussation in the brainstem. Ultimately, it aids in looking down and out [3].

Parinaud syndrome, also known as dorsal midbrain syndrome, is often attributed to pressure on the pretectal region within the brain, most commonly by a pineal gland mass, leading to this rare syndrome. Bienfang from UpToDate (Wolters Kluwer Health, Waltham, MA) reports that this syndrome includes vertical gaze diplopia that worsens with upward gaze, preference for downward deviation of the side of the lesion, downbeating nystagmus, and bilateral lid retraction [4]. In a study of 206 children with pineal gland tumors, the most common abnormal finding was abnormal pupil size. However, 180 of the patients had limited vertical gaze and diplopia. The most common etiology was compression hydrocephalus [5]. Additionally, a case report discussed by Bhola et al. identified a patient with chronic vertical diplopia secondary to a cerebrovascular accident two years prior. The cause was attributed to a brainstem hemorrhage extending from the pontomesencephalic junction with physical exam findings including vertical gaze palsy, diplopia, and blurred vision [2]. 

While Parinaud syndrome remained on the differential, the patient was solely having difficulty with vertical gaze and associated diplopia, making a CN IV palsy more likely. These findings on examination are subtle and can easily be missed. Cornblath identifies one approach for acute diplopia. First, the physician must differentiate between monocular and binocular diplopia. If only one eye is affected, retinal emergencies are far more likely. However, if there is binocular diplopia, a central source of the deficit increases in likelihood. It is then important to assess for singular versus multiple CN involvement which is dependent on a thorough CN examination. Furthermore, additional focal deficits must be assessed. Ultimately, when these steps are followed correctly, appropriate disposition can be streamlined with appropriate communication with consultants [3]. Lee et al. discuss two cases where the microvascular disease was the culprit for the neuropathy. They reported that, generally, the symptoms present abruptly and improve over time, consistent with the presenting case here. While MRI may show infarction in the midbrain, it is not well demonstrated along the fourth nerve and is presumptive rather than confirmative [6].

## Conclusions

Although CN IV neuropathy is not a diagnosis that is the final responsibility of the ED physician, using a multifaceted approach with a basic understanding of anatomy and pathophysiology can allow for better history acquisition and more thorough physical examinations. Ultimately, this allows for well-informed, appropriate consultation and resource utilization when acute diplopia presents to the ED.
